# Game Algorithm of Intelligent Driving Vehicle Based on Left-Turn Scene of Crossroad Traffic Flow

**DOI:** 10.1155/2022/9318475

**Published:** 2022-09-09

**Authors:** Zonghuan Guo, Dihua Sun, Lin Zhou

**Affiliations:** ^1^School of Automation, Chongqing University, 174 Shazheng Street, Shapingba District, Chongqing, China; ^2^Dongfeng Xiaokang Automobile Co., Ltd, No. 1 Jiujiang Avenue, Shuangfu New District, Jiangjin District, Chongqing, China

## Abstract

Vehicle networking and autonomous driving are hot areas of scientific research today, and they complement each other and play an important role in people's intelligent travel. Intelligent driving vehicle can enhance road safety, effectively reduce traffic flow and fuel consumption, and promote the overall social development. It has great application value in urban traffic system. The traffic condition of a city directly affects the economic development of the city and the improvement of people's quality of life. As the “core” of the urban traffic network, intersections are the frequent places where traffic jams occur. Game theory, as a win-win theory, mainly solves the problem of multiperson and multi-objective with contradictory objective functions and can be used to study the optimal signal control strategy. Aiming at this problem, the potential conflict behaviors of intelligent driving vehicles when turning left at urban intersections are analyzed and a decision model is established. A long-term trajectory prediction model of straight vehicles is established based on the Gaussian process regression model (GPR) considering the vehicle motion pattern. Combined with trajectory prediction, a decision-making process (model) for intelligent driving vehicles based on conflict resolution and a multifactor driving action selection method are proposed. A coordination algorithm based on game theory is designed for conflicting vehicles. The proposed algorithm is verified by the self-developed intelligent vehicle hardware simulation platform. The simulation results show that the PID method based on digital identification and positioning makes the intelligent vehicle obtain good system step response, can improve the disturbance tracking ability of intersection turning analysis, meet the requirements of turning control system, and reduce the complexity and randomness of parameter design, which is better than the traditional fuzzy control method.

## 1. Introduction

Intelligent driving will greatly improve production efficiency and traffic efficiency, and may become the first breakthrough field of artificial intelligence. Intelligent driving will be an important branch of solving traffic congestion in the future, which can greatly improve production efficiency and traffic efficiency. On the one hand, with the popularization of intelligent driving, traffic congestion is no longer a problem. People can accept longer commuting distance. Cars can be a natural extension of homes and offices, which is more conducive to the construction of new urbanization. On the other hand, the operation of intelligent driving vehicles requires supporting transportation infrastructure. Due to intelligent driving, Zhang sensor senses road obstacles, or communicates with road facilities through 4g/DSRC. Therefore, guide cables, magnetic sign columns, radar reflective signs, sensors, communication facilities, etc., need to be arranged at intersections, roadsides, bends, etc. Traditional means of transportation, such as cars, are the representative products of industrial civilization, while artificial intelligence is the representative product of information society. The combination of the two is intelligent driving, which is an important representative of the integration of industrialization and industrialization. Therefore, intelligent driving is not only a new generation of transportation but also a terminal and interactive platform for personalized needs and data collection. It is also the core link of the new intelligent manufacturing system and industrial value chain. Intelligent driving plays an important role in ensuring road traffic efficiency and traffic safety [1]. Under the dynamic urban environment, especially at urban intersections, the behavior decision-making research of intelligent vehicles is one of the key factors that restrict their real road driving [2]. Therefore, it is of great practical significance and academic value to strengthen the research on traffic decision-making at urban intersections. The intelligent vehicle control system is based on digital identification and positioning, and performs operations such as direction identification, information feedback, central control, and mechanical execution [3]. The sampling statistics of traffic accidents in domestic cities show that the traffic accident rate at grade intersections is about 30%. Therefore, finding an effective intersection coordination control method is of great significance to improve intersection traffic efficiency, reduce intersection traffic accidents, and improve the overall traffic safety level [4]. The driving site of the intelligent vehicle is complex. Through the modules of location, information identification, and steering operation, the driver can be assisted to drive along the preset path, and the information of the intersection can be identified and judged to realize the correct turn. The turning control system is the technical key to the normal running of intelligent vehicles, and it is also the execution basis of the internal analysis of steering gear and the external output of steering wheel [5].

In 1963, computer technology was applied to the traffic signal control system for the first time, which greatly improved the performance and level of the traffic control system and became a milestone in road traffic signal control technology [6]. Due to the significant correlation between the self-weight of the smart car, the steering gear power module and the steering gear response, and the large delay of the steering gear itself, it is impossible to build an accurate steering gear steering control model [7]. As the intersection is a common place where traffic accidents occur, autonomous vehicle must consider how to pass through this complex scene safely and effectively [8]. With the continuous evolution of safety technology and the continuous expansion of safety dimensions and boundaries, automatic driving safety currently mainly includes several dimensions. The first is basic safety, which we call the cornerstone of safety, which can ensure the basic safety ability of automatic driving. The second dimension is functional safety, which is the connotation of safety. It ensures that automatic driving has the ability to deal with the failure or failure of system software and hardware, and reasonably control risks. The third dimension is the expected functional safety, which is the expectation of safety, to ensure that autonomous driving has the ability to deal with hazards and hazard risks caused by nonfault scenarios such as system limitations, environmental impact, and personnel misuse. The last dimension is network information security, which is a new dimension of security to ensure that the auto-drive system has the ability to deal with network attacks, information leakage, and other harmful risks. However, due to the randomness and development of urban traffic conditions, the existing intersection control strategies cannot fully meet the needs of urban traffic. Moreover, although many control strategies have made theoretical breakthroughs, it is not mature to apply them to urban intersections to solve the problem of road congestion [9]. Therefore, it is of great practical significance to deeply study the state characteristics and control characteristics of urban intersections, and constantly explore and develop new control ideas and concepts [10]. The driving behavior decision-making algorithm is the technical core and the focus of technological breakthroughs in smart cars. The research on the driving behavior decision-making algorithm of smart cars can effectively improve the driving safety of smart cars, reduce the occurrence of traffic accidents, and improve people's travel experience. It will greatly promote the development of autonomous driving technology and related technology industries. Through the use of vehicle communication, vehicle road communication, and other technologies to achieve information interaction, this greatly reduces the perceived pressure and computing time cost of autonomous vehicle at intersections.

This paper presents a multivehicle cooperative control algorithm based on game theory. The innovation of this algorithm is that it has been verified on the independently developed hardware simulation platform of intelligent vehicle. In the algorithm selection module, the multiperson cooperative game theory is applied to the intersection signal control as a new control strategy. By analyzing the adaptability of the control method in the field simulation software under different traffic levels, the best applicable conditions of this control method are obtained. The simulation results show that the PID method based on digital recognition and location makes the intelligent vehicle obtain good system step response. It can improve the interference tracking ability of intersection steering analysis, meet the requirements of steering control system, and reduce the complexity and randomness of parameter design. This is better than the traditional fuzzy control method. When traffic conflicts occur at intersections, speed control can prevent collisions, avoid parking, and improve traffic efficiency.

## 2. Related Work

Under the complex and dynamic time-varying traffic environment, the decision making of intelligent driving vehicles is closely related to the motion state of traffic participants. Many scholars have carried out research on intelligent behavior decision based on the prediction-decision framework. Cao et al. proposed a lane-changing behavior prediction model based on dynamic Bayesian network (DBN) in combination with vehicle status, road structure, workshop interaction, and other characteristic attributes, and verified it on the public data set [11]. Li et al. proposed a prediction method of lane-changing behavior based on deep learning. In this method, the lane-changing scene data of vehicles on the expressway are transformed into a special format of top view pictures, which are input into the recurrent neural network (RCNN) for training, and a more accurate prediction model is obtained without explicitly establishing the vehicle interaction relationship [12]. Cheng et al. provided reference for the strategy selection of driverless vehicles by analyzing the differences between the driving intentions and their expected driving actions of vehicles that have potential conflicts with the vehicle, and setting different levels of risk early warning in combination with the intersection structure [13]. Zheng et al. used gap acceptance theory and lane change decision rules to build a free lane change decision model [14]. Huang used RSAN (rough-set artificial neural network) to study the decision making in car-following scenarios [15]. Zheng et al. combined with the state grid method to plan cubic and quartic polynomial curves. And the parameter curve in the interpolation curve planning algorithm based on Bezier curve needs to be controlled? We define its shape, and the core of Bezier curve is Bernstein polynomial [16]. Song et al. analyzed the game characteristics and game models existing in the traffic signal control at the intersection, analyzed the game characteristics in the multiphase signal control of the single intersection in detail, studied the method of multiplayer cooperative game in the multiphase signal control of the single intersection, and established the corresponding game model and solved [17]. Zheng et al. introduced the overall architecture of the system. After introducing the working principle of each module in the system, the information transmission relationship between each module is given [18]. In order to predict the behavior of intelligent vehicles to other vehicles at intersections, Dewangan and Sahu designed finite state machine models for straight-ahead and turning-through intersections, and then combined with safety judgment rules, and realized the safe passage of intelligent vehicles at intersections [19]. Li et al. simulated the control method in the second simulation software and analyzed the applicable conditions of the control method in the single-intersection multiphase signal control through the obtained evaluation data [20].

In this paper, vehicle sensors and camera methods are used to collect traffic data at typical urban intersections, and vehicle motion patterns are recognized. Then, based on the Gaussian process regression model, the vehicle is modeled and long-term predicted. Finally, a left turn decision model based on conflict resolution theory is established and its effectiveness is verified.

## 3. Methodology

### 3.1. Intelligent Car Intersection Left Turn Control System

**Figure 1:**
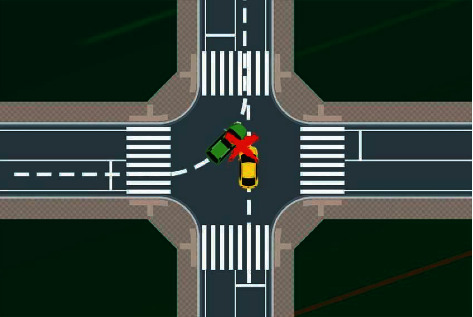
Schematic diagram of the intersection.

The turning of intelligent vehicles at intersections requires the help of digital identification and positioning to obtain the path information, which is transmitted to the steering gear processor to complete the turning operation. Based on the application of digital recognition and positioning technology to track and adjust the intelligent vehicle, intelligent driving uses internal model control function and fuzzy PID algorithm for reference. Fuzzy PID control method is used to complete the construction of intelligent vehicle intersection turning control system. The accuracy and rapidity of its recognition ensure the safety of vehicles. There are multiple conflict points at the intersection. Here, we take the conflict point 1 between the west to east vehicle *V*₁ and the south to north vehicle *V*₁ as an example, and the analysis results are also applicable to other conflict points at the intersection.  Figure 1 shows the schematic diagram of a simple two-way two-lane intersection without traffic light control.

**Figure 2:**
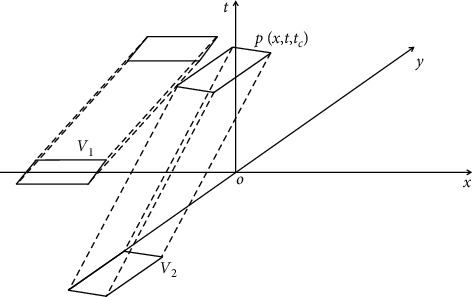
VTF of vehicles in conflict at intersection.

Assume that *V*₁ and *V*₁ in Figure 1 are straight vehicles, and their speed, acceleration, and distance from the conflict point are Ʊ₁, *a*₁, *l*₁, and Ʊ, respectively. There are 4 conflict points, that is, ⑨, *a* Э, *l* Э, 1, 2, 3, and 4. When *V* and *V* pass through the intersection, there are two situations: there is a traffic conflict in the workshop, and keeping the current driving speed will lead to the collision between the two cars. There is no traffic conflict in the workshop, so the two cars will not collide at the current speed. To judge whether the two cars are in a traffic conflict state, the field map tool is introduced to analyze the conflict judgment process as shown in Figure 2.

In the figure, *xoy* coordinate plane is the projection of the vehicle on the ground, including the shape and size of the vehicle; axis *t* is the time axis, and the origin of axis *t* is the current time. If the field graph is used to describe the future motion trajectory of the vehicle, it is called virtual trajectory field (VTF). Here, the VTF method is used to predict the future motion trend of vehicle 2 and judge the conflict state. When designing the coordination algorithm, it is necessary to determine the first decision-making time point Tb and the last decision-making time point Te of the vehicles at the intersection. Here, Tb is taken as the time point when the vehicles first discover the conflicting vehicles after entering the intersection, and Te is taken as the time point when the conflicting relationship between the two vehicles ends.

To turn a smart car at an intersection, it needs to use digital identification and positioning to obtain path information, and transmit it to the steering gear processor to complete the steering operation. The steering gear is an important component of the turning control system, including turntable, mechanical gear set, position locator, steering drive motor, and intelligent integrated circuit board, which mainly adjusts the turning angle through continuous control signals. Turning lag is due to the longtime of identification, transmission, and analysis of positioning information. Therefore, it is necessary to improve the analysis efficiency of turning and reduce the impact of lag time on turning effect. In the process of turning, effectively reducing unnecessary damage will fundamentally improve the overall production efficiency. And through the reasonable selection of cutting tools, the frequency of adverse phenomena in the turning process can be reduced. And it can also play a good management role in the cost of the workpiece. Only by continuously improving the use of equipment in production and processing can the overall production efficiency be fundamentally improved. However, there is a linear relationship between the pulse signal width and the angle of the steering gear, and it is necessary to add a first-order plus pure lag link between them to adjust the angle difference, which is calculated as:

(1) $$\begin{array}{c} G\left(s\right)=\frac{\theta }{l{\left(Ts+1\right)}^{•e^{-ts}}}\text{,} \end{array}$$

where *G* (*s*) is the steering transfer function; *T* pulse transmission time; and *τ* is the steering hysteresis parameter.

**Figure 3:**
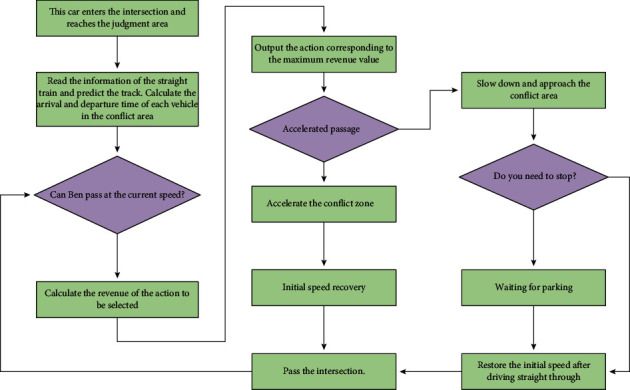
Action selection process in the single-vehicle scene.

When the intelligent driving vehicle enters the intersection, it predicts the trajectory of the straight vehicle and obtains the time interval for passing through the conflict area. If the two may meet in the conflict area, the left-turn vehicle needs to choose an appropriate speed and control the vehicle to avoid the straight-going vehicle in time. In the actual simulation application process, the vehicle speed is controlled by the PID module, and the acceleration and deceleration actions are generated according to the current vehicle speed and the input expected vehicle speed. The decision-making process under the single vehicle scenario is obtained by comprehensively considering the action selection, efficiency, comfort, and other factors, as shown in Figure 3.

### 3.2. Traffic Control Model

The lateral stability of the vehicle through the intersection is ensured by adding dynamic constraints. After the optimal control sequence at the current moment is calculated, it is input into the controlled platform, and then, the platform executes the received control instructions and then inputs the observed value of the state quantity at the current moment into the state estimator. In addition, the state estimator can also estimate the state quantities that cannot be observed directly (such as the ground adhesion coefficient). Finally, the state variables are input to the MPC controller for the next optimization solution, and the control variables required by the controlled platform at the next time are obtained. From this, we can get the strategic thinking of crossing: for the risk of collision caused by only crossing routes of vehicles in other directions, we can adjust the time of arriving at the crossing by adjusting the speed of self-driving vehicles, so as to avoid collision; for vehicles in other directions whose final routes coincide with our own vehicles, we can adjust the control strategy of self-driving vehicles based on longitudinal safety to achieve the purpose of collision avoidance. The application of the Internet of Vehicles helps us to identify the driving intentions of other vehicles and also enables self-driving vehicles to pass through the crossing more safely and efficiently.

In the multilane traffic flow model, the relationship between traffic flow and vehicle density and vehicle speed is as follows:

(2) $$\begin{array}{c} \rho \left(t+1,i,l\right)=\rho \left(t,i,l\right)+\frac{T}{L_{i}}\left\{q\left(t,i-1,l\right)-q\left(t,i,l\right)+u\left(t,i,l\right)-e\left(t,i,l\right)\right\} \end{array}$$

(3) $$\begin{array}{c} v\left(t,i,l\right)=x_{f}b_{i}\exp\;\left[-0.5{\left(\frac{\rho \left(t,i,l\right)}{\rho_{cr}}\right)}^{2}\right]\text{,} \end{array}$$

(4) $$\begin{array}{c} q\left(t,i\right)=a\rho \left(t,i\right)v\left(t,i\right)+\left(1-a\right)\rho \left(t,i+1\right)v\left(t,i+1\right)\text{.} \end{array}$$

Assuming a one-way two-lane expressway, Section 1 has vehicles changing from Lane *l* to Lane *y* and no vehicles changing from Lane *y* to Lane *L* in time period *tT*, (*t*+1), and it is easy to know that the density of the two lanes at time *t* + 1 of equation (2) at *μ*_*yl*_(*t*, *i*)=0 is

(5) $$\begin{array}{c} \rho \left(t+1,i,l\right)=\rho \left(t,i,l\right)+\frac{T}{L_{i}}\left[q\left(t,i-1,l\right)-q\left(t,i,l\right)+u\left(t,i,l\right)-e\left(t,i,l\right)\right]-\eta_{ty}\left(t,i\right)\rho \left(t,i,l\right)\text{.} \end{array}$$

By (3), the speed of the two lanes at the moment is

(6) $$\begin{array}{c} v\left(t=1，i，l\right)=v_{f}b_{i}exp\left[-0.5{\left(\frac{\rho \left(t+1,i,l\right)}{\rho_{cr}}\right)}^{2}\right]\text{.} \end{array}$$

According to the relationship between traffic flow and density and speed, there are

(7) $$\begin{array}{c} q\left(t+1,i,l\right)=\rho \left(t+1,i,l\right)v\left(t+1,i,l\right)\text{.} \end{array}$$

The definition *I*[*η*_*ty*_(*y*, *i*)] is that in the time period [*tT*, (*t* + 1)*T*], there is a vehicle change from the *l* lane to the *y* lane and there is no vehicle change in the *y* lane.

When reaching the *l* lane, the total flow of the two lanes at time *t* + 1 is, namely:

(8) $$\begin{array}{c} I\left[\eta_{ty}\left(t,i\right)\right]=q\left(t+1,i\right)=1\left(t+1,i,l\right)+q\left(t+1,i,y\right)\text{.} \end{array}$$

On the basis of ensuring safety conditions, it is necessary to select actions with the shortest total time to ensure the efficiency of the traffic process, namely:

(9) $$\begin{array}{c} action=act_{c\left(\;\min\right.\left(T_{action}\right)}\text{,} \end{array}$$

where *T*_action_ is the value of *t* and *t* that takes less time, to show that choosing this time represents a more efficient action to execute.

Through the above derivation, the optimization problem represented by formula (5) is transformed into the standard form of the quadratic programming optimization problem represented by formula (6), and the optimal future control variable sequence at the current moment can be obtained by solving it. Then, the first control variable of the sequence is used as the actual control variable to act on the system to control it, and as time goes by, continuous prediction, rolling optimization, and control are carried out. Nonlinear model predictive control (NMPC) and linear time-varying model predictive control (LTV-MPC) are analyzed, and the linearization steps of nonlinear model predictive control model are derived in detail. Then, the LTV-MPC predictive control model is derived in detail. Finally, the steps of transforming multiconstraint LTV-MPC problem into quadratic programming (QP) problem and its subsequent solution process are derived in detail. Through the analysis of the intersection scene, the safety constraints are added to the motion planning of the autonomous vehicle, and then, the vertical and horizontal coupling motion control algorithm of the autonomous vehicle is designed and studied. Considering the complex environment when the vehicle is actually driving, the above constraints alone are not enough. For example, if a vehicle encounters a bad working condition in which pedestrians or vehicles suddenly appear in front of it during driving, the driver needs to take emergency braking or hit the steering wheel to avoid collision. In this case, the risk coefficient will further increase with the decrease of the ground adhesion coefficient. On the other hand, the rear axle of the vehicle will skid, resulting in an irreversible jerking phenomenon. Therefore, by adding the dynamic constraints of the vehicle, the stability requirements of the autonomous vehicle in the trajectory motion planning and control process can be further met.

The motion planning layer is responsible for calculating the safe, comfortable, and dynamic feasible trajectory of the target configuration provided by the behavior layer from the current configuration of the vehicle to the decision-making level. The target configuration may vary depending on the environment. The target location can be the center point of the current lane, that is, a few meters in front of the direction of travel, the center of the stop line at the next intersection, or the next desired parking space. The motion planning component receives information about static and dynamic obstacles around the vehicle and generates a collision-free trajectory, which meets the dynamic and kinematic constraints on vehicle motion.

## 4. Result Analysis and Discussion

In order to fully consider the possible dangerous scenes that autonomous vehicle may encounter at intersections, from the perspective of the number of traffic participants and the way the vehicle passes through the intersection (straight or turning), the full array combination method is used to obtain all basic dangerous collision scenes without signal lights, and then, all scenes without collision conflict with the main vehicle are eliminated or simplified. According to this principle, we can get all the dangerous scenes that eventually collide with the main vehicle. After obtaining the dangerous scene database of intersections with and without signal lights, the new motion planning and control framework and control algorithm of autonomous vehicles proposed above are used for simulation verification. Hazard identification is the driver's subjective understanding of the potential hazards of objective traffic, so the level of identification is different and can be improved through continuous learning. Through the analysis of the front-end driving risk prediction, it is the premise to avoid the danger in the process of driving with foresight. We consider dangerous traffic scenarios with more than two traffic participants, as shown in Table 1.

Table 1 lists the scenarios without traffic lights with traffic participants 2 and 3 according to the number of traffic participants, starting positions and routes, and then proposes or simplifies the scenarios that do not conflict with the host vehicle. The simulation in the previous section shows that the proposed new motion planning and control framework for autonomous vehicles can well complete the traffic task, and this control logic is still applicable when the number of traffic participants is more than 4. For intersections with signal lights, it is only necessary to eliminate the scenes that will not occur according to the traffic rules to obtain the dangerous scene library. However, most intersections with signal lights are large intersections. Although the probability of danger is much smaller than that of small intersections without signal lights, the consequences of accidents will be more serious due to the fast speed. Therefore, it is also necessary to conduct simulation tests on these dangerous scenes.

**Figure 4:**
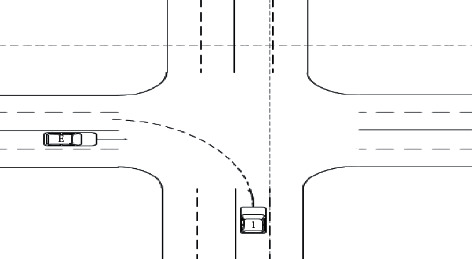
Schematic diagram of left turning conditions of vehicles in other directions.

The vehicle *E* is the self-driving main vehicle, and the vehicle 1 is the interference vehicle. At the beginning of the simulation, the main vehicle *E* is 40 m away from the center of the intersection and runs along the center line of this lane at a speed of 21.6 km/h, while the interfering vehicle 1 is 22 m away from the center of the intersection. It goes straight into the intersection at a constant speed of 14.4 km/h and then turns left. The host car *E* receives the lane keeping instruction from the upper-level driving behavior planning module, and at the same time, in order to avoid a collision with the interfering car arriving at the intersection at the same time, the host car starts to slow down and yield to the interfering car 1 to let it go first, and then accelerates to pass the intersection. The simulation conditions are shown in Figure 4.

**Figure 5:**
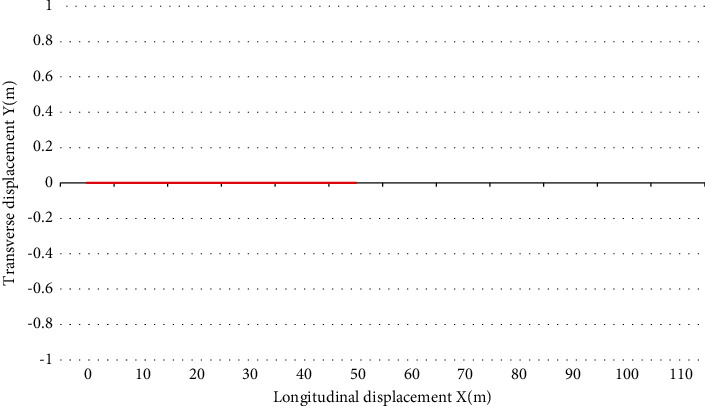
Left turn of vehicles in other directions—time domain response.

**Figure 6:**
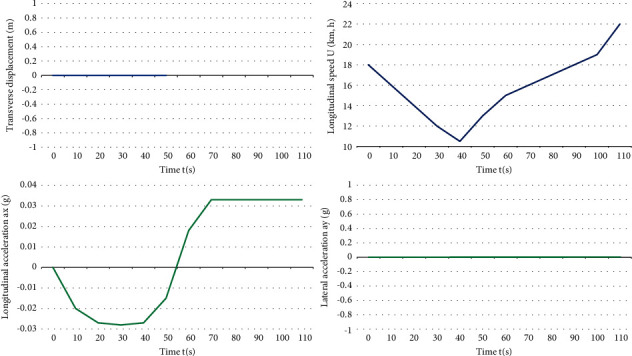
Left turn of vehicles in other directions—time domain response.

It can be seen that after receiving the driving state information of the interfering vehicle 1, it is known by calculation that there is a risk of collision between the interfering vehicle 1 and the host vehicle *E*, and the host vehicle *E* gives the interfering vehicle 1 permission by appropriate deceleration courtesy, so that the host vehicle *E* slows down and delays the arrival time at the intersection to avoid collision with the interfering vehicle 1. During the whole simulation process, the main vehicle *E* and the interference vehicle 1 did not have any collision, the absolute value of the actual longitudinal acceleration was less than 0.1 g, and the riding comfort was good as shown in Figures 5 and 6.

It can be seen that after receiving the steering command, it is judged by calculation that there is a danger of collision between the host car and the interfering car 1, and the host car *E* starts to slow down and politely give way to the interfering car 1. By adjusting the speed, the host car *E* and the interfering car 1 are staggered to arrive at the intersection, and after steering, the host car follows the interfering car 1 and continues to drive forward. During the whole simulation process, the main vehicle *E* and the interference vehicle 1 did not have any collision, the absolute value of the actual longitudinal acceleration was less than 0.1 g, and the riding comfort was good.

**Figure 7:**
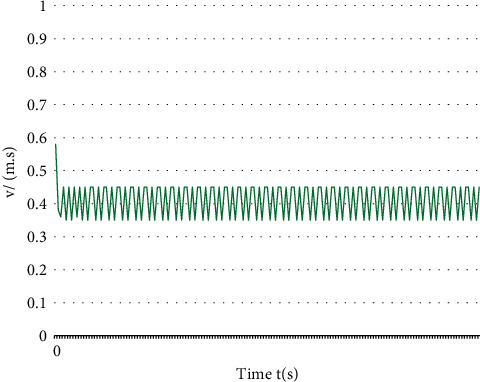
Actual speed of hardware simulation.

**Figure 8:**
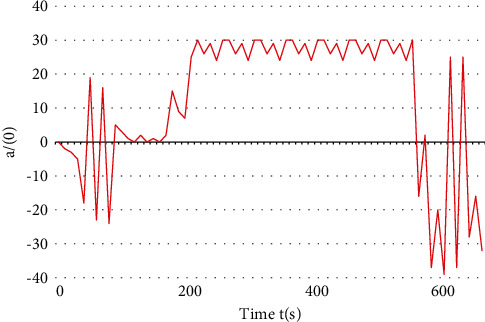
Hardware test angle command.

It can be seen from the experimental results that the proposed algorithm can avoid the traffic conflict through speed control. Using the conflict table algorithm requires the vehicle to stop at the intersection and wait for the conflict vehicle to pass through the intersection. This algorithm can make the vehicle pass through the intersection earlier, improve the traffic efficiency of the intersection, and reduce the energy consumption in the process of vehicle starting as shown in Figures 7 and 8.

The model built with biped can use the footstep animation tool to realize the movement of the vehicle. According to the motion path and speed of the bus and car, the appropriate hardware test angle of the model is estimated, and the key points are created to realize the motion analysis. As the actual hardware experiment eliminates the wireless control of the upper computer, it has better real-time control effect, smoother corner command, and more stable speed control. The designed intelligent car simulation platform has the following characteristics: A. Software simulation can simulate the state of LC resonant sensor in real electromagnetic field environment to some extent, and it has the characteristics of low cost and short development cycle. B. Hardware simulation can observe and record the car body state and control effect in real time, which is convenient to track the dynamic running process of the program and provides a good platform for debugging complex algorithms.

## 5. Conclusions

This paper presents a multivehicle cooperative control algorithm based on game theory. In the algorithm selection module, the multiperson cooperative game theory is applied to the intersection signal control as a new control strategy. By analyzing the adaptability of the control method in the field simulation software under different traffic levels, the best applicable conditions of the control method are obtained. This paper analyzes the decision making of left turn behavior of intelligent vehicles at urban intersections in the intersection scene. Gaussian process model is used to predict the trajectory of oncoming through vehicles, and a left turn decision model is established based on conflict resolution theory. The conclusions are as follows: (1) according to the classification of five motion modes of vehicles at urban intersections, Gaussian process model is used to predict the long-term trajectory of through traffic. Finally, the high-precision long-term prediction trajectory, which is very close to the actual situation, is obtained; (2) in the algorithm selection module, the multiperson cooperative game theory is applied to the intersection signal control as a new control strategy. By analyzing the adaptability of the control method in the field simulation software under different traffic levels, the best applicable conditions of the control method are obtained; (3) the behavior of turning left to cross the intersection is divided into three stages: crossing motor vehicles, nonmotor vehicles, and pedestrians. The simulation results show that the PID method based on digital recognition and location makes the intelligent vehicle obtain good system step response. It can improve the interference tracking ability of intersection steering analysis, meet the requirements of steering control system, and reduce the complexity and randomness of parameter design. This is better than the traditional fuzzy control method. When traffic conflicts occur at intersections, speed control can prevent collisions, avoid parking, and improve traffic efficiency. However, the experimental results lack real database validation, and the samples are too small. Therefore, the algorithm can avoid traffic conflict through speed control, and further verification and analysis are needed.

## Figures and Tables

**Table 1:** Dangerous scenes at crossroads.

	Liangche		Sanche	
	Same road car	Different cars	Same road car	Different cars
The main car goes straight	Cut out the front car	Other obstacle cars cut in, and the main car goes straight	Cut out the front car	In the other direction, two obstacle cars follow, and the main car politely or passes through
	Distant pursuit	Other obstacle cars go straight or turn, and the main car goes straight		The main car follows the car from a long distance, and other directions interfere with the car passing through
	Front emergency stop			The main car and the interfering car are in two different lanes, and the main car goes straight

Main steering	The car goes straight ahead, and the main car turns	The main car turns, and the other obstacle cars go straight and turn		The front car goes straight, the main car turns, and other obstacles go straight or turn
	The front car turns with the main car	After the main vehicle turns, the vehicle in front of the lane moves slowly		The main car and the two obstacle cars have two different lanes, and the main car turns

## Data Availability

The raw/processed data required to reproduce these findings cannot be shared at this time as the data also form part of an ongoing study.
